# “Who needs secure services for personality disorder?” Results of an expert Delphi study with professional staff

**DOI:** 10.1186/s12888-019-2268-3

**Published:** 2019-09-10

**Authors:** Zoe Foyston, Celia Taylor, Mark Freestone

**Affiliations:** 1 Manchester University NHS Foundation Trust, Manchester, UK; 2 East London NHS Foundation Trust, London, UK; 3 Centre for Psychiatry, Queen Mary University of London, Charterhouse Square, London, EC1M 6BQ UK

**Keywords:** Personality disorder, Offenders, Health services, Pathway, Delphi, Consensus, Suitability

## Abstract

**Background:**

Personality Disorder (PD) is an enduring, multi-faceted mental disorder, associated with adverse health effects, difficulties with interpersonal relationships and in some cases increased risk to others. A limited number of dedicated forensic mental health services are available for serious offenders with severe personality disorder. The recent Offender Personality Disorder (OPD) strategy aims to ensure that most such offenders are treated in prison rather than secure psychiatric services, except in highly complex cases where this is not possible. While the strategy sets out very broad criteria relating to this, greater clarity is needed to support decisions about appropriate transfer and hence enhance public protection. This study explored which characteristics professional experts associate with appropriate transfer from prison to forensic mental health services for high-risk offenders with PD.

**Method:**

A modified Delphi survey distributed through an online survey system was conducted in two-rounds with a group of professional experts recruited from forensic mental healthcare; criminal justice and specialist commissioning.

**Results:**

Fifty-one (56%) respondents completed stage one of the Delphi and 34 (61%) of these completed stage two. Consensus was reached for a total of 22 items indicating complexity, including co-morbid mental illness, high level of risk, lack of progress in prison and high motivation for treatment. A preliminary checklist for these factors was developed. Panel members consistently emphasised the importance of the individual’s presenting need, the overall clinical picture and formulation in their free text responses.

**Conclusions:**

Professionals face a complex picture when making decisions regarding suitability for hospital admission for high-risk male offenders with PD, with varied opinions amongst professional experts as to priorities for intervention and a focus on individual needs through formulation. It was, nevertheless, possible to condense these views into a set of consistent variables that can be used to highlight the need for transfer into hospital-based treatment services.

**Electronic supplementary material:**

The online version of this article (10.1186/s12888-019-2268-3) contains supplementary material, which is available to authorized users.

## Background

Personality Disorder (PD) is a recognised, multi-faceted mental health disorder [1] that is characterised by persistent, problematic and pervasive patterns of behaviour that deviate markedly from the expectations of the individual’s culture [2]. People with a diagnosis of PD have complex needs, due to the heterogeneity of the presentation of the disorder [3], and the fact that PD is commonly co-morbid with Axis I mental disorders, substance abuse [4] or other PDs [5]. Epidemiological studies have suggested that between 4 and 10% of the adult population of the UK lives with a PD [6, 7]. PD is associated with early mortality [8], psychiatric morbidity [9] self-harm and violence [10], higher rates of unemployment [6], and decreased quality of life [11]. The prevalence of PD has been shown to be significantly higher in the Criminal Justice System compared to community samples [12], with potentially up to70% of offenders affected [13]. The resource consequences for the health care and criminal justice systems are considerable, and the impact on society is disproportionate [14].

Historically, there has been a deficiency in the health service provision for individuals with PD [15], with inpatient beds in the UK overwhelmingly occupied by people with severe and enduring mental illness [16]. Currently, there is limited consensus and guidance in the literature about what constitutes progress in treatment and how this should be measured [17, 18].

### Treatment of PD in forensic mental health settings

Offenders with PD are still considered one of the most difficult groups to treat, with many studies reporting engagement difficulties, high rates of attrition [19], and poor psychosocial and clinical outcomes [20]. Individuals that commence treatment but disengage prematurely have been shown to have a greater risk of recidivism compared to those who never started treatment in the first place [21]. Therefore, it is important that offenders with PD receive treatment best suited to their needs, risk and presentation.

Prior to 2015, most services for offenders with PD were concentrated in high and medium secure hospitals under the Dangerous and Severe Personality Disorder (DSPD) programme [22]. However, in 2015 those resources were reorganised in accordance with the new Offender Personality Disorder (OPD) pathway strategy. The OPD strategy was commissioned to improve identification and assessment of offenders with PD, providing psychologically informed services for the most high-risk and complex cases [23]. It aims to offer a holistic approach, considering an offender’s journey, from conviction and sentencing through to community re-settlement, and proposing a coherent and consistent transition between the specialist criminal justice and health services. One of the main aims of the OPD strategy was ensure many more offenders with PD were treated in prison settings, with transfer to hospital reserved only for those unable to benefit from this.

### Efficient management of scarce resources

Estimates suggest that 20,000 offenders in England and Wales meet the OPD criteria [24]. However, the associated costs for treating offenders with PD are high [25], and treating these individuals in a specialised forensic mental health service is much more expensive than a standard prison programme. For example, it is estimated that it costs £175,000 (approx. USD $222,000) to detain a patient in a Medium Secure Unit per annum [26]; in contrast, detaining an offender in a Male Category B prison costs an estimated £25,007 (approx. USD £31,700) per patient per annum [27].

Therefore, a key rationale of the move to the OPD Pathway was the very low cost-effectiveness of the hospital services contained within the DSPD Programme, which offered a very small number of beds with a very high monetary investment to prevent few future offences [28]. Establishing specialist prison-based services enables many more high-risk PDOs to receive interventions within a more efficient setting; this also implies that decisions to transfer an individual to a specialist forensic mental health service need to be based upon sound evidence and professional consensus. However, the question of what constitutes appropriate transfer from prison to hospital has never been clarified. The OPD pathway sets out very broad, non-exclusive criteria such as co-morbid severe mental health problems, added neurological difficulties, uncertain or disputed diagnosis and high risk [29]. Some of these criteria also lack a clear rationale, with many individuals meeting these criteria serving their sentence in a prison environment. Existing evidence suggests that currently, decision-making within teams working with offenders with a PD diagnosis is inconsistent, inequitably applied – e.g. less often to ethnic minority cases – and can even tend to undermine the institutional purpose of these hospital services [30]. This is likely because, in the UK the legislation covering hospital transfer was.

### Multi-disciplinary working

Multi-disciplinary working and specialist training are considered critical for success in working with offenders with PD [31]. The OPD strategy highlights the importance of joint operations and collaboration between prison, probation and secure mental health services [32]. Thus, within OPD pathways services, support and advice are provided by clinicians working alongside probation officers, and that a formulation and pathway plan are completed jointly [23]. This means clinicians and practitioners who play an integral role in the assessment and treatment of offenders with PD are often responsible for the decision to make a referral to a health pathway. To date, the perspectives of professionals with expertise in the field have been absent from empirical research regarding this matter, despite a recent literature review identifying the potential negative impacts of working with this offender group (Freestone et al., 2015). Due to the fundamental role they play, capturing the perspectives of these individuals is fundamental to developing coherent and consistent practice.

A systematic review of the literature [33] indicated several common characteristics of individuals with PD transferred to health pathways, as well as identifying predictors, mediators and moderators of successful treatment. Whilst the systematic review yielded 38 relevant papers, no article clearly identified the characteristics associated with appropriate and successful transfer from prison to a hospital setting. Therefore, greater clarity is needed. This study aims to address this gap in the literature by consulting directly with senior professionals practising within the OPD pathway to obtain consensus regarding the factors associated with a need for medium and high secure psychiatric services for male offenders with PD.

## Method

A modified Delphi Survey [34] was conducted in two rounds. The Delphi method is an anonymous, iterative process that aims to obtain expert consensus on a complex problem through a process of convergence of ideas and solutions. A two-round Delphi process was selected to minimise attrition, reduce the influence of group opinions on the individual, and to ensure the survey was informed by the current evidence base [35].

### Participants

Participants were professional experts or published researchers in the field who were affiliated to one of three professional domains: forensic mental healthcare; criminal justice and specialist commissioning. Researchers were identified through a systematic review of the literature with corresponding authors contacted. Clinicians were identified through a snowballing process beginning with established working relationships with the study team, as well as searching clinical service directories. Electronic snowballing then ensued, with those responding asked to nominate any other individuals they thought would meet the eligibility criteria.

To be eligible in the study individuals needed to be:

- Registered or qualified (i.e., PhD, MD, DClinPsych, DipSW, RCN or HCPC registered) professional staff.
- Aged 25 or over; to ensure that respondents had a degree of post-qualification clinical or professional experience at the time of responding.
- Substantively employed within, or involved in the commissioning of, a PD service.

Delphi surveys typically yield response rates of approximately 66–82% [36], meaning a larger number of professional experts were contacted than the required sample. We aimed to recruit equal numbers of professional experts from each domain.

### Procedure

A 37-point Likert scale Delphi survey (Additional file 1) was developed using the SurveyMonkey web package. The question content was derived from a systematic review of the literature [30] and items were classified into four themes: diagnosis; clinical characteristics; offence history; and ‘other’. The survey was piloted by two independent professionals. Jairath and Weinsten [37] emphasise the importance of pilot testing to identify any errors or administration difficulties.

An initial email was sent to professional experts in the field outlining the study, detailing the inclusion criteria, gauging interest and requesting agreement to participate. Individuals who agreed to participate were emailed a link to access the questionnaire via the SurveyMonkey website (www.surveymonkey.com). A copy of this initial questionnaire is available as Additional file 1. On activating the link, participants had a total of 14 days to complete the survey. A reminder email was sent to participants if they had not completed the survey within 7 days.

In round one, participants were asked to rate the importance of characteristics of offenders with PD across three outcome dimensions:

1. Suitability for hospital admission
2. Successful outcome of treatment e.g. reduction in symptoms, distress and/or psychopathology
3. Likely to be a risk factor in future re-offending

Participants could respond via a 5-point Likert rating scale (highly likely, somewhat likely, unsure, somewhat unlikely, highly unlikely), and were able to insert free-text comments or qualifying remarks. Participants were also asked to comment on the following additional factors: level of risk, the impact of the therapeutic relationship, important staff qualities and the optimum length of stay.

The results from the first round were analysed and used to create the second-round survey, and participants who had consented to be contacted again were emailed a link to the second-round survey. In the second round, participants were presented with feedback on the responses of the panel as a whole - in this case the percentage of panellists who suggested a specific item would be suitable for hospital admission– and were asked to re-rate the item using the same Likert scale as in previous rounds.

### Ethics

Ethical approval for this study was obtained by the North Central London Research Consortium, NoClor), the NHS Local Ethics service, the NOMS Research Approval Committee and the UK Health Research Authority (HRA). The reference for the Ethical Approval is 16/WS/0004.

For both rounds, written consent was obtained electronically, and participants were informed of their right to withdraw at any stage. To ensure anonymity, participants were administered a unique, eight-digit identification number which allowed the researcher to identify their data should the individual decide to withdraw from the study.

### Analysis

First-round responses were analysed to provide an initial breakdown of the frequency of endorsed items (n, %). The five-point Likert scale attached to each item was condensed into two categories: ‘Likely’ combining the ‘highly likely’ and ‘somewhat likely’ responses; and Unlikely/Unsure combining the ‘highly unlikely’, ‘somewhat unlikely’ and ‘unsure’ responses. The ‘Unsure’ responses were pooled with the ‘Unlikely’ responses because this was interpreted by researchers as a failure to positively endorse an item. Consensus of an item was defined as equal to or greater than 50% agreement of the ‘Likely’ responses. Free-text responses were analysed using thematic analysis and any additional items absent from the initial survey were highlighted. Items mentioned repeatedly under any item in the survey and endorsed by more than 20% of individuals were also included in the second-round survey.

For the second round, items from the original questionnaire with > 50% endorsement, or new items with > 20% endorsement were included in a revised questionnaire, and respondents were asked to re-rate the new items in the list on the same Likert scale. Those items exceeding 50% consensus were included in the final list of factors, and then used as the basis of a draft screening questionnaire for admission to PD services.

## Results

Forensic services for personality disorder in the UK is a new and relatively specialised field, which comprises approximately 1760 beds in total across the mental health and criminal justice systems [38]. Based on a review of the literature and personal contacts, as well as the service directory maintained by the UK prison and probation service [39] we developed a matrix of services, and identified a population of 120 experts, clinicians, researchers and commissioners who linked to these services or active in the field of forensic personality disorder treatment or research. we believe the original population was representative of expertise in England and Wales at the time of the study. During round one, 91 people agreed to participate in the study with 63 respondents (69%) opening and entering the survey: 51 (56%) completed the survey in full; 8 (9%) partially completed it and four (4%) did not progress past entering their unique identification number. Of the 51 completed participants, four (8%) asked not to be contacted regarding the second-round survey.

For round two, the survey was sent to all 59 consenting panel members with a total of 36 (61.01%) individuals opening and entering the survey: 34 (57.62%) completed this in full and two (3.38%) partially completed it. The overall response rate was 61% which is in line with other Delphi studies [36]. Of the total sample, respondents were psychologists, psychiatrists, psychotherapists, researchers, commissioners, occupational therapists, clinical leads and nurses. Table 1 displays the occupational breakdown of panel members for each round of the survey. The panellists completing both rounds were from health (17; 47.22%), the criminal justice system (14; 38.89%), commissioning (2; 5.56%), a research background (2; 5.56%), and one (2.78%) individual whose sector of work could not be identified.

**Table 1:** Occupational breakdown of panellists

	n	%
Round 1		
Psychologist	30	47.61
Psychiatrist	16	25.40
Psychotherapist	5	7.94
Researcher	3	4.76
Commissioning	3	4.76
Occupational Therapist	2	3.17
Nurse	1	1.59
Other or did not disclose	3	4.76
Round 2		
Psychologist	15	41.67
Psychiatrist	9	25.00
Psychotherapist	2	5.56
Researcher	2	5.56
Commissioning	2	5.56
Occupational Therapist	2	5.56
Nurse	1	2.78
Other or did not disclose	3	8.33

### Round 1

Consensus (50% endorsement or better) was reached for 23 (66%) of the items regarding suitability of hospital admission: 5 (14%) from diagnosis, 8 (22%) from characteristics, 8 (22%) from offence history and 6 (16%) from other. Two items from offence history were merged (previous convictions; early onset of offending) due to their similar content, meaning a total of 22 items were included in the second-round survey. The highest consensus item was co-morbid PD & Severe and enduring Mental Illness (SMI) with 100% of experts indicating suitability for hospital admission. Ambiguity arose from one of the items (IQ), meaning further clarification was required during the second-round survey. Eleven items (31%) failed to meet the 50% threshold, meaning these items were discarded and omitted from the second-round survey.

### Results of round 2

During round-two consensus was reached for 22 (81.48%) of the 27 items. Table 2 displays the number and percentage of endorsed items in ranked order. PD & SMI remained the most frequently endorsed item with 34 (94.44%) individuals indicating suitability for hospital admission. Despite the high number of items endorsed by participants throughout, panel members consistently emphasised the importance of the individual’s presenting need, overall clinical picture and formulation in their free text responses.

**Table 2:** Heat map of experts’ rating for all items listed in round-two in ranked order

Items	Likely	Unlikely/Uncertain	Consensus?
PD + SMI^a^	94.44%	5.56%	Yes
Psychiatric History	91.67%	8.33%	Yes
Co-morbid PD	86.11%	13.89%	Yes
BPD	83.33%	16.67%	Yes
High Motivation	83.33%	16.67%	Yes
Self-Harm	82.86%	17.14%	Yes
Suicidal Ideation	77.78%	13.89%	Yes
Lack of Progress in Prison	77.14%	22.86%	Yes
PD + MI^b^ Not Severe	75%	25%	Yes
Need for Further Assessment	71.43%	28.57%	Yes
PPD	69.44%	30.55%	Yes
Poor Social Functioning	69.44%	30.56%	Yes
MI Managed by Medication	68.57%	31.43%	Yes
Interpersonal Aggression	66.67%	33.33%	Yes
High Risk	65.71%	34.29%	Yes
Very High	65.71%	34.29%	Yes
Previous Trauma	62.86%	37.14%	Yes
Prison - Previous Psych Treat	60%	40%	Yes
Impulsivity	55.56%	44.44%	Yes
Offence History	51.43%	48.57%	Yes
Any other Psych Treatment	51.43%	48.57%	Yes
Hostility	50%	50%	Yes
10+ Convictions	45.71%	54.29%	No
LD	45.71%	54.29%	No
Age 35<	44.44%	55.56%	No
Sexual Index Offence	40%	60%	No
Medium Risk	37.14%	62.86%	No

^a^*SMI* Severe and enduring mental illness

^b^*MI* Mental illness

**Fig. 1:**
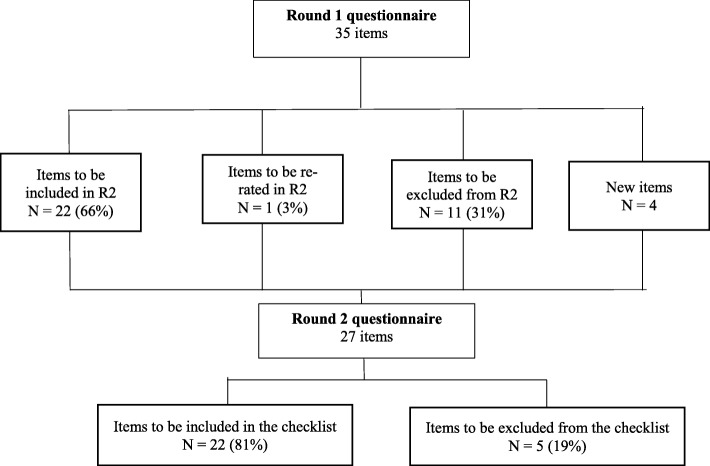
Flowchart displaying the number and outcomes for each item in the first round of the Delphi survey

Five items (18.52%) failed to reach consensus: having had ten or more convictions prior to the age of 18 (45.71%); individuals with learning difficulties (45.71%; age (44.44%; sexual index offence (40%) individuals deemed medium risk of violence (37.14%). The iterative process of developing the final checklist is detailed in Fig. 1.

### Diagnosis

During round two, all five of the included diagnostic categories reached consensus among participants (PD & SMI, 94.44%; co-morbid PDs, 86.11%; borderline D, 83.33%; PD & SMI not severe and enduring, 75%; paranoid PD, 69.44%). Co-morbid presentations were endorsed more frequently than diagnosis alone, with participants accentuating that hospital admission should be reserved for those individuals with complex presentations:

*“Related to complexity and severity [and therefore] worthy of higher resourced hospital unit.”*

*“Again, [co-morbid presentations] likely give [the] perception that such clients are more complex and risky, therefore more likely to need hospitalisation”*

*“[T]he more complex presentations may require admission simply for better assessment and understanding; however I would still expect the vast majority of people could [then] be managed / treated in prison as well as they could be in hospital.”*

PD and co-morbid SMI was the most frequently endorsed category with 94.44% of respondents reporting suitability for hospital admission. Panel members emphasised the occurrence of psychotic symptoms as the important feature, rather than the PD diagnosis itself. Of the diagnoses alone, BPD reached the highest consensus 83.33%, with participants highlighting the difficulty of assessing and managing risk, as well as the increased resource demands in cases of repeated self-harm and the impact this had on staff working with this diagnosis:

*“[It] depends with how client is presenting. I think the anxiety generated by working with this client group generates more anxiety in staff teams that [elicit] feelings of powerlessness that possibly lead to compensate with hospitalization”*

*“I think it would be wrong to assume that all service users would need to be presenting with BPS traits to the detriment of others who do not. They are definitely a key client group requiring PD services who also create the highest rate of staff burn out.”*

### Clinical characteristics

During round two, seven (87.5%) of the eight included characteristic items reached consensus (psychiatric history, 91.67%; motivation, 83.33%; suicidal ideation, 77.78%; poor social functioning, 69.44%; interpersonal aggression, 66.67%; impulsivity, 55.56%; hostility, 50%). Previous psychiatric history including a past diagnosis of SMI or a previous inpatient psychiatric admission (91.67%) was the most frequently endorsed item, with experts suggesting the outcome of previous admissions needed consideration.

Suicidal ideation (77.78%) was the second most endorsed item with panellists reinforcing the need to assess risk in their free-text responses. Respondents linked hospital admission to acute risk and short-term placements aimed at reducing the immediate risk of harm:

*“It is easier to observe someone in hospital and the environment tends to be safer, in addition short term pharmacological interventions can be helpful. I think these admissions should be focussed and shorter term as part of an overall pathway though.”*

*“Maybe appropriate where there is acute risk.”*

An offender’s age was the least frequently endorsed characteristic, with only 44.44% of panel members indicating this was an important characteristic when considering hospital admission.

### Offence history

For round two, two of the offending items were merged – early onset offending and having ten or more convictions prior to the age of 18 – because of their similar content. This meant three items linked to offence history were included. Previous offence history was the only item to reach consensus (51.43%), with panel members rather divided in their responses. Experts indicated in their free-text responses that offence history alone would not necessarily be sufficient to determine hospital admission, highlighting the need to consider the severity and nature of the offending history, the diagnosis and presentation, taking into consideration other clinical factors and the formulation:

*Relevant but not a determining factor. May bring secondary information around impact of previous interventions which would be relevant*

*Each time around needs to be assessed case by case to remain curious as to what's changed or what the problem is.*

### Other

During round one, panellists were asked what level of risk they thought was most suited for treatment in a hospital setting: low, medium, high or very high-risk individuals. The results suggest experts feel hospital admission should be reserved for those individuals who pose a significant risk to others; endorsement rates for this factor were medium risk (60.71%), high risk (86.25%) and very high risk (72.55%), meaning all three of these items were re-ranked in the second-round survey. In the second round, survey participants reached consensus on both the high and very high risk categories but failed to do so for medium risk individuals. This confirms that professional experts believe that hospital admission should be reserved for the most high-risk cases.

During round two participants again reached consensus on mental illness managed by medication (68.57%); previous psychological treatment received in prison (60%), attendance at any other psychological programme (51.43%) and mental illness managed by medication (66.67%). Free-text responses relating to engagement with previous psychological programmes emphasised the importance of considering the individual’s engagement and outcome of treatment with experts split on what this meant for hospital admission:

*Depends on progress / gain rather than ‘completion’ although not dropping out would be a positive marker*

*It may be indicative of motivation, progress but admission to hospital services should be made based on current presentation in my view.*

Additionally, panellists were asked to comment on the importance of the therapeutic relationship in treatment success, 100% of respondents indicated the significance of this when offenders received care in the hospital setting. Respondents emphasised the significance of the therapeutic relationship, suggesting this underpinned the process, considered to be more important than treatment modality:

*My understanding of the literature is that regardless of intervention methods the therapeutic alliance is one of the most important factors. My clinical experience also supports this*

*Therapeutic relationship has the biggest impact on treatment outcomes irrespective of treatment modality.*

### Newly identified items

Experts were given the opportunity to identify any additional characteristics associated with suitability for hospital admission not included in the first-round survey. Experts generated a total of 32 additional items, and items with two or more endorsements are displayed in Table 3. The responses were reviewed and evaluated by the researchers to prevent duplication and to ensure they were in line with the research question.

**Table 3:** Newly generated items in ranked order

Characteristics	Sum of n	Percentage (%)	Included in second round survey
Lack of progress in prison	31	61	Yes
Self-harm	19	37	Yes
LD - IQ below 75	15	29	Yes
Trauma	11	22	Yes
Disputed diagnosis/further assessment	10	20	Yes
Levels of subjective distress	6	12	No
Severity of condition/symptoms	6	12	No
Medication required	5	10	No
Support network	3	6	No
Emotional dysregulation	3	6	No
Length of sentence	3	6	No
Intimate partner violence	2	4	No
ASD	2	4	No
Treatment resistant illness	2	4	No
Institutional care	2	4	No
Physical health needs	2	4	No
LGBT (Transgender)	2	4	No
Ability to build relationships	2	4	No
Frequent management and segregation	2	4	No
Introverted	2	4	No

Five items were endorsed by more than 20% (*n* = 10) of individuals across the survey and were incorporated in to the second-round survey: lack of progress in prison; self-harm; LD (IQ score below 70); progress in previous treatment; trauma. The first-round-survey included a characteristic item relating to IQ (below 90), however participants expressed lower IQ scores (below 70) were perhaps more suitable for hospital admission. Further clarification relating to IQ was required, therefore the original IQ item was revised to incorporate LD (IQ score below 70). This means a total of four additional items were included in the subsequent round and one item (IQ) required further clarification.

All four newly generated items reached consensus in round two: self-harm (82.86%); lack of progress in prison (77.14%); need for further assessment (71.43%); and previous trauma (62.86%). Self-harm was the highest endorsed item: panel members expressed the view that hospital admission might be applicable to help stabilise patients and reduce risk. Interestingly, participants utilising the free text box highlighted that this alone should not warrant a hospital admission:

*Should not be a reason for admission to hospital: forensic or not! Should be trigger for active treatment though*

*May indicate need for secure environment. Other factors determine more if FMH [forensic mental health] is appropriate.*

The item relating to individuals with LD did not reach consensus. (45.71%). There was acknowledgment from participants that this population are likely to require specialist services, however, forensic mental health services might not be the most beneficial:

*Depends on services: most MSUs can't manage them and the staff are not used to them; it causes all sorts of problems.*

*Yes, to a specialist LD service. This way the staff have additional training and can be responsive to the service users’ needs. It also provides the service users with a better opportunity for progression, given they cannot access mainstream offending behaviour treatment, placing them at a disadvantage to other service users.*

### Prototype screening checklist

The final step of this study was to combine the identified and endorsed items from Round Two of the Delphi into a checklist to screen for suitability for potential transfer to forensic mental health services. Those items which received more than 50% endorsement by experts were included into the checklist, which was divided into three key areas: diagnosis, clinical characteristics; and prior history and engagement (see Table 4). Internal Reliability scores for the three areas were acceptable, with Cronbach α = .863 for the diagnosis scale, α = .836 for the clinical scale and α = .826 for the progression scale.

**Table 4:** Prototype screening checklist for appropriate transfer of offender with personality disorder to forensic mental health services

D	Diagnostic Considerations	Yes	No
D1	Presence of personality disorder (PD) comorbid with severe and enduring mental illness.	+ 1	0
D2	PD comorbid with mental illness not considered severe or enduring e.g. mood disorder.	+ 1	0
D3	Is the mental illness managed appropriately by medication?	0	+ 1
D4	More than one PD diagnosis.	+ 1	0
D5	Diagnosis of borderline personality disorder.	+ 1	0
D6	Diagnosis of paranoid personality disorder	+ 1	0
C	Clinical Characteristics		
C1	Offender has repeated incidents (2+) of self-harm.	+ 1	0
C2	Offender presents an acute risk of suicide.	+ 1	0
C3	Offender is engaging in verbal aggression, abuse or threats to other individuals.	+ 1	0
C5	Offender assessed as being at high or very high risk of violence.	+ 1	0
C6	Offender has a history of previous trauma	+ 1	0
C7	Offender presents with impulsivity	+ 1	0
C8	Offender has poor social functioning.	+ 1	0
C9	Evidence of identified need for further assessment of offender.	+ 1	0
P	Prior Treatment History and Engagement		
P1	Successfully attended and engaged in previous psychological programmes either in prison or in the community setting?	+ 1	0
P2	One or more convictions aged less than 18 years?	+ 1	0
P3	Motivation to engage in treatment.	+ 1	0
P4	Lack of progress in the prison setting?	+ 1	0
T	Total Score		

The checklist requires external validation and calibration (i.e., what is the threshold for admission) but could serve in the meantime as a guide to factors that clinicians should consider when an offender who may potentially benefit from transfer to forensic services is first identified. This questionnaire could be readily validated against existing data on offenders transferred into Forensic Mental Health services and calibrated against successful onward transfers from these services.

## Discussion

This study aimed to identify those characteristics associated with successful transfer from prison to forensic mental health services for high-risk male offenders with PD, via a systematic consultation exercise with professional experts.. Participants reached consensus on 22 (60%) of the items, which is in line with most other Delphi studies. However, it is important to note that our consensus rate was defined at 50% endorsement which is lower than other articles in the literature.

### Key consensus items

Co-morbidity items were endorsed more frequently than PD diagnoses alone, reflecting professional experts’ view that hospital settings should be reserved for the most complex cases where co-morbidity is a significant issue. Additionally, participants’ responses highlighted that the type of PD was an important consideration, with BPD and PPD deemed more suitable for transfer to the hospital setting. Likewise, acute risk of suicide and repeated self-harm were also clinical characteristics requiring consideration, emphasising the importance of assessing the severity and risks posed. Individuals deemed to pose a ‘low-risk’ of violence was the least frequently endorsed item, which is in line with the OPD pathway criteria as it excludes men who are low-risk of harm, despite at times having equally severe personality difficulties [22]. What is also clear is that the consensus around diagnostic suitability is relatively weak, implying that this issue is not considered to be of prime importance. Each case requires careful thought and consideration, with professionals emphasising the importance of the overall clinical picture and formulation.

Prior treatment history and engagement were thought to be highly significant when making decisions about an individual’s pathway. Factors such as lack of progress in prison, and the individual being motivated, were considered relevant by panellists when considering suitability for hospital admission and successful treatment outcome but were not considered risk factors in future re-offending. The universality of offenders’ motivation as a driver of efficacy highlights the importance of focussing on it throughout their custodial sentence. Interventions that target engagement, commitment and PD symptomatology, such as the ‘goals-based approach’ [40] have shown promise and are already utilised in some clinical settings. Similar models could be employed across an offender’s journey from sentencing to community re-settlement.

Decisions regarding hospital admission are also complex because they typically involve several professionals from a variety of different agencies in the UK: the NHS, the prison and probation services, commissioners and the Ministry of Justice [22] A considerable degree of heterogeneity was observed between professional experts’ responses across the different items, with many items not reaching the 50% consensus threshold. If professionals have such varied and diverse opinions regarding this subject matter, it is legitimate to question how consistently the broad guidance currently given in the Strategy translates in practice. This study has been able to extract those factors about which there is some consensus, and which can provide a structured, informed approach to making referrals to forensic mental health services. This study therefore not only reinforces the importance of clear decision-making criteria, but also of collaborative working across the distinct parts of the system [23, 32].

### Strengths of this study

The Delphi method is an anonymous, iterative process that aims to obtain expert consensus, over a complex problem through a process of convergence. This method is advantageous as it gathers expert opinion where lack of clarity exists, across dispersed geographical areas [41]. Similarly, it protects participant anonymity, which encourages the free expression of views. The construction of the Delphi was derived from a systematic review of the literature, ensuring its content was grounded in previous research and adding validity to the results. Since many of the critical decisions about care pathways and suitable services for PDOs within the CJS are made by the very group of professionals sampled, the study offers a unique insight into the emerging consensus within this population. Where such a population is available, Delphi and similar methods are often used to develop clinical guidelines under conditions of uncertainty [42] including NICE guidance [43].

Surowiecki [44] suggested the following are required to ensure the robustness of group findings: diversity of expertise; independence; decentralisation; and aggregation. The current study adhered to these principles, ensuring a heterogeneous sample of experts from health, criminal justice and commissioning, encompassing diversity of job role and title. Many Health Service and CJS decisions rely on professional opinion born of years of training and experience. Experts completed the survey independently, reducing the effects of bias due to group interactions. Finally, the methods of aggregation were decided in advance, thus reducing the influence of researcher bias. All panel members had a wealth of experience and professional training, providing relevant and thoughtful free-text responses which further add to the strength of the findings.

### Limitations of this study

Professional consensus is subjective in nature, based entirely on opinion which has the potential for error. Critics of the Delphi method suggest it lacks in scientific rigour [45], and is, or should be, categorised at the bottom of the hierarchy of evidence [46]. The invitation to participate in the survey was sent to over 100 experts, with only a proportion expressing interest and completing the survey. It has been suggested that those who participate are likely to be impacted upon by the outcome of the process [39], which in turn suggests that commitment to the process could well reflect an individual’s own interest in the subject matter. This could, in theory, indicate a biased sample.

The Delphi study had a modest sample size, with a high number of individuals from health and the criminal justice system, with a small, albeit proportional, number of commissioners (8%). Similarly, panel members were predominately professionals working and practising in England, meaning the generalisation of results to, for example, Wales should be taken with caution.

Additionally, the process of aggregation in the Delphi study is open to arbitrary judgement [40] with studies defining consensus in a variety of ways. While this degree of subjectivity allows for freedom within a research project, it does impact on what is reported. For this study, 50% endorsement was required, however other studies have set this figure higher e.g. Hart et al. [47] set theirs at 90% and Berk et al. [37] at 80%. However, in an area where consensus is lacking and likely to be controversial, this figure can be lower and in some cases below 50% (e.g. [48]). In this case, a lower endorsement rate of 50% was preferred for two reasons: i) due to the lack of an established evidence base for the effectiveness of services for personality disorder, determined from the literature; and ii) to ensure that a breadth of opinion was captured by the survey and no potentially important factors omitted. This was on the premise that items could subsequently be tested empirically and retained or discarded.

## Conclusions

We conducted an expert Delphi study where we asked professionals with experience of working with offenders with a likely diagnosis of personality disorder for their opinions on the factors that related to suitability of high-risk offenders for transfer to secure health services. The professionals considered these services to be most suitable for offenders with comorbid DSM Axis I and Axis 2 mental disorders; a prior history of psychiatric hospitalisation; co-occurring personality disorders; or borderline personality disorder. They also reached a broad consensus that self-harming behaviour, a high motivation to engage in treatment, and a lack of progress in prison were important factors indicating suitability. However, the level of overall agreement on several other items was below 75%, suggesting a breadth of opinion, which was corroborated by the range of free-text comments given by respondents emphasising formulation, clinical judgement and an individualised approach to offenders’ care.

Future research should seek to test the factors identified in this Delphi to explore their relationship with successful health transfer and subsequent treatment; this analysis could be conducted with research data or high-quality routine clinical data.

## Additional file

Additional file 1: Electronic Delphi Questionnaire utilised at stage 1 of the data collection detailing the initial items reviewed by participants. (PDF 782 kb)

## Data Availability

The datasets used during the current study are available from the corresponding author on reasonable request.

## Abbreviations

CJS: Criminal Justice System
DSPD: Dangerous and Severe Personality Disorder
NHS: UK National Health Service
OPD: Offender Personality Disorder Pathway
PDO: Offender with a diagnosis of personality disorder

## References

[1] American Psychiatric Association (2013). Diagnostic and statistical manual of mental disorders.

[2] Kaplan HI, Sadock BJ (1998). Sudy guide and self-examination review for Kaplan and Sadock’s synposis of psychiatry.

[3] Gibbon S, Duggan C, Stoffers J, Huband N, Vollm BA, Ferriter M, Lieb K (2010). Psychological interventions for antisocial personality disorder. Cochrane Database Syst Rev.

[4] Grant BF, Stinson FS, Dawson DA, Chou SP, Ruan WJ, Pickering RP (2004). Co-occurrence of 12-month alcohol and drug use disorders and personality disorders in the United States: results from the national epidemiologic survey on alcohol and related conditions. Arch Gen Psychiatry.

[5] Zimmerman M, Rothschild L, Chelminski I (2005). The prevalence of DSM-IV personality disorders in psychiatric outpatients. Am J Psychiatry.

[6] Coid J, Yang M, Tyrer P, Roberts A, Ullrich S (2006). Prevalence and correlates of personality disorder in Great Britain. Br J Psychiatry.

[7] Samuels J (2011). Personality disorders: epidemiology and public health issues. Int Rev Psychiatry.

[8] Chesney E, Goodwin GM, Fazel S (2014). Risks of all-cause and suicide mortality in mental disorders: a meta-review. World Psychiatry.

[9] Bennett A, Johnson D (2017). Co-morbidity of personality disorder and clinical syndrome in high-risk incarcerated offenders. J Forensic Pract.

[10] Haw C, Hawton K, Houston K, Townsend E (2001). Psychiatric and personality disorders in deliberate self-harm patients. Br J Psychiatry.

[11] IsHak WW, Elbau I, Ismail A, Delaloye S, Ha K, Bolotaulo NI, Nashawati R, Cassmassi B, Wang C (2013). Quality of life in borderline personality disorder. Harv Rev Psychiatry.

[12] Fazel S, Danesh J (2002). Serious mental disorder in 23000 prisoners: a systematic review of 62 surveys. Lancet.

[13] Ministry of Justice and Department of Health U (2011). Working with personality disordered offenders: a practitioners guide.

[14] Dixon-Gordon KL, Whalen DJ, Layden BK, Chapman AL (2015). A systematic review of personality disorders and health outcomes. Can Psychol.

[15] National Institute for Mental Health in England (2003). Personality disorder: no longer a diagnosis of exclusion.

[16] Melzer D, Tom B, Brugha T, Fryers T, Gatward R, Grounds A, Johnson T, Melzer T (2004). Access to medium secure psychiatry care in England and Wales. 1: a national survey of admission assessments. J Forensic Psychiatry Psychol.

[17] National Institute for Health and Care Excellence UK (2009). Antisocial personality disorder: prevention and management.

[18] National Institute for Health and Care Excellence UK (2009). Borderline personality disorder: recognition and management.

[19] Fortune Z, Barrett B, Armstrong D, Coid J, Crawford M, Mudd D, Rose D, Slade M, Spence R, Tyrer P (2011). Clinical and economic outcomes from the UK pilot psychiatric services for personality-disordered offenders. Int Rev Psychiatry.

[20] Greeven PG, De Ruiter C (2004). Personality disorders in a Dutch forensic psychiatric sample: changes with treatment. Crim Behav Ment Health.

[21] McMurran M, Theodosi E (2007). Is treatment non-completion associated with increased reconviction over no treatment?. Psychol Crime Law.

[22] DSPD Programme (2008). Dangerous and severe personality disorder (DSPD) high secure services for men: planning and delivery guide.

[23] Benefield N, Joseph N, Skett S, Bridgland S, D'Cruz L, Goode I, Turner K (2015). The offender personality disorder strategy jointly delivered by NOMS and NHS England. Prison Serv J.

[24] National Health Service England (2015). The offender personality disorder pathway strategy.

[25] Barrett B, Byford S, Seivewright H, Cooper S, Duggan C, Tyrer P (2009). The assessment of dangerous and severe personality disorder: service use, cost, and consequences. J Forensic Psychiatry Psychol.

[26] Walker J, Amos T, Knowles P, Batson S, Craissati J (2012). Finance. Putting a price on psychiatric care. Health Serv J.

[27] Ministry of Justice UK (2016). Costs per place and costs per prisoner by individual prison: national offender management service annual report and accounts 2015–16 management information addendum.

[28] Barrett B, Tyrer P (2012). The cost-effectiveness of the dangerous and severe personality disorder programme. Crim Behav Ment Health.

[29] Joseph N, Benefield N (2012). A joint offender personality disorder pathway strategy: an outline summary. Crim Behav Ment Health.

[30] McRae L (2013). Admitting offenders with antisocial personality disorder to a medium secure unit: a qualitative examination of multidisciplinary team decision-making. J Forens Psychiatry Psychol.

[31] Ramsay M (2011). The early years of the DSPD (dangerous and severe personality disorder) programme: Results of two process studies. Ministry of Justice research reports.

[32] Logan C, Ramsden J (2015). Working in partnership: making it happen for high risk personality disordered offenders. J Forensic Pract.

[33] Freestone M, Munholland E, Foyston Z, Taylor C (2018). Identifying characteristics associated with appropriate transfer to forensic mental health services for male offenders on the offender personality disorder pathway (OPDP).

[34] Linstone HA, Turoff M (2002). The Delphi method: techniques and applications.

[35] Dalkey NC (1972). Studies in the quality of life; Delphi and decision making.

[36] Akins RB, Tolson H, Cole BR (2005). Stability of response characteristics of a Delphi panel; application of bootstrap data expansion. BMC Med Res.

[37] Jairath N, Weinstein J (1994). The Delphi methodology (part one): a useful administrative approach. Can J Nurs Adm.

[38] McRae L (2016). Severe personality disorder, treatment engagement and the legal aid, sentencing and punishment of offenders act 2012: what you need to know. J Forens Psychiatry Psychol.

[39] HM Prison and Probation Service and NHS England (2019). Brochure of offender personality disorder services for men.

[40] McMurran M, Jinks M (2012). Making your emotions work for you: a pilot brief intervention for alexithymia with personality-disordered offenders. Personal Ment Health.

[41] Hasson F, Keeney S, McKenna H (2000). Research guidelines for the Delphi survey technique. J Adv Nurs.

[42] Black N, Murphy M, Lamping D, McKee M, Sanderson C, Askham J, Marteau T (1999). Consensus development methods: a review of best practice in creating clinical guidelines. J Health Serv Res Policy.

[43] National Institute for Health and Care Excellence (2014). Developing NICE guidelines: the manual.

[44] Surowiecki J (2004). The wisdom of crowds: why the many are smarter than the few.

[45] Sackman H (1974). Delphi critique: expert opinion, forecasting and group process.

[46] The Joanna Briggs Institute Levels of Evidence and Grades of Recommendation Working Party (2014). Supporting document for the Joanna Briggs Institute levels of evidence and grades ofrecommendation.

[47] Hart LM, Jorm AF, Kanowski LG, Kelly CM, Langlands RL (2009). Mental health first aid for indigenous Australians: using Delphi consensus studies to develop guidelines for culturally appropriate responses to mental health problems. BMC Psychiatry.

[48] Cohn DE, Havrilesky LJ, Osann K, Lipscomb J, Hsieh S, Walker JL, Wright AA, Alvarez RD, Karlan BY, Bristow RE (2015). Consensus in controversy: the modified Delphi method applied to gynecologic oncology practice. Gynecol Oncol.

