# Supplementary material for: “Who needs secure services for personality disorder?” Results of an expert Delphi study with professional staff
Source: BMC Psychiatry. 2019 Sep 10;19:280. doi: 10.1186/s12888-019-2268-3 (PMC6734324; doi:10.1186/s12888-019-2268-3)
Supplement: Supplementary file 1 — Electronic Delphi Questionnaire utilised at stage 1 of the data collection detailing the initial items reviewed by participants. (PDF 782 kb) [file 12888_2019_2268_MOESM1_ESM.pdf]

Assessing Offenders Transfers from Prison to Forensic Mental Health Services

Consent Form

In this study we aim to review healthcare professional's assessment of male offenders transferred from correctional settings to hospital forensic mental health services on the Offender Personality Disorder Pathway (OPD). This study is being completed by Dr Mark Freestone and Zoe Foyston from Queen Mary University of London, Barts & The London School of Medicine and Dentistry, Wolfson Institute of Preventative Medicine, Centre of Psychiatry.

Thank you for agreeing to take part in our study. We are looking into the characteristics that professionals believe are relevant when deciding whether an offender with personality disorder needs to be transferred from prison to hospital for further assessment and/or treatment. This will allow us to infer which individuals cannot be treated in prison, and require health-based interventions in order to maximise engagement and positive outcomes.

You have been chosen to take part in the survey because of your experience and expertise in the field. The statements you are about to see relating to suitability for treatment in hospital have been derived from a systematic review of the literature and we do not necessarily endorse any of these. This is a Delphic process that will be lead by your opinion, so please be as honest and forthright as you can.

Your participation in this research study is entirely voluntary and you may withdraw at any time. We will do our best to keep all information provided confidential by storing this in a password protected format. To help protect your confidentiality, the survey will not contain any information that will personally identify you. You will be asked to enter the eight digit identification number that was sent in your email invitation before beginning the survey. This number will be used by the researchers to identify any data you provide if you decide to withdraw at any point.

Please note, that by clicking 'Next' you are consenting to allow the answers you provide to be utilised in our research, and that by completing the survey you agree to complete the follow-up survey, once all answers from this round have been synthesised. This survey will be part of a two-stage Delphic process and at the end of this round we will summarise the results in terms of the factors viewed as most important in a hospital transfer and contact consenting participants to comment further on the summary.

The following questions should not cause you distress or discomfort. However, if there are any issues that you would like to discuss, or you wish to withdraw your data at any point, please feel free to contact Zoe Foyston, Researcher, at z.n.z.goodwin@smd16.qmul.ac.uk

This survey will take most people approximately 30-40 minutes to complete. Thank you again for participating in our study your feedback is invaluable.

Assessing Offenders Transfers from Prison to Forensic Mental Health Services

Identification Number

To protect your confidentiality please enter the eight digit identification number located in your invitation. This number will be used to identify participants if they decide to withdraw..

* 1. Unique identification number

* 2. Please enter your job role and speciality:

Contents Page

- **Personality Disorder Diagnoses: Page 4 - 5**
- **Co-morbid Diagnoses: Pages 6 - 7**
- **Characteristics: Pages 8 - 11**
- **Offence History: Pages 12 - 13**
- **Other: Pages 14 - 17**

Diagnoses

As you will be aware, there are numerous different diagnoses of Personality Disorder. As part of this survey we wish to further understand how each disorder responds to treatment, helping us to gain further insight into what diagnoses are more responsive to treatment in a hospital setting, thinking about what works and with whom.

In this section we will ask your thoughts on a number of Personality Disorder diagnoses: ASPD; EUS: BPD; PPD; DPD; Psychopathy, asking you to consider each diagnosis across three different domains: 1) Suitability for hospital admission 2) Successful treatment outcome in a hospital setting (i.e. reduction in symptoms, distress and/or psychopathology) 3) Reduction in the likelihood of re-offending
We acknowledge there are large individual differences between each patient and within each disorder, however for the purpose of this study we ask that you think holistically.

* 1. Antisocial Personality Disorder (ASPD):

	Highly likely	Somewhat likely	Somewhat unlikely	Highly unlikely	Unsure
Suitability for hospital admission	○	○	○	○	○
Successful treatment outcome in hospital setting	○	○	○	○	○
Reduction in likelihood of re-offending	○	○	○	○	○

Please add your comments, provisos and/or qualifying remarks

2. Are there specific interventions that in your experience, are particularly effective in a hospital setting with offenders who have a diagnosis of ASPD? (e.g. psychoeducation, Cognitive Behavioural Therapy, etc.)

* 3. Emotionally Unstable: Borderline Personality Disorder (BPD):

	Highly likely	Somewhat likely	Somewhat unlikely	Highly unlikely	Unsure
Suitability for hospital admission	○	○	○	○	○
Successful treatment outcome in hospital setting	○	○	○	○	○
Reduction in likelihood of re-offending	○	○	○	○	○

Please add your comments, provisos and/or qualifying remarks

4. Are there specific interventions that in your experience, are particularly effective in a hospital setting with offenders who have a diagnosis of BPD? (e.g. psychoeducation, Cognitive Behavioural Therapy, etc.)

Diagnoses

* 1. Paranoid Personality Disorder (PPD):

	Highly likely	Somewhat likely	Somewhat unlikely	Highly unlikely	Unsure
Suitability for hospital admission	◯	◯	◯	◯	◯
Successful treatment outcome in hospital setting	◯	◯	◯	◯	◯
Reduction in likelihood of re-offending	◯	◯	◯	◯	◯

Please add your comments, provisos and/or qualifying remarks

2. Are there specific interventions that in your experience, are particularly effective in a hospital setting with offenders who have a diagnosis of PPD? (e.g. psychoeducation, Cognitive Behavioural Therapy, etc.)

* 3. Dependent Personality Disorder (DPD):

	Highly likely	Somewhat likely	Somewhat unlikely	Highly unlikely	Unsure
Suitability for hospital admission	◯	◯	◯	◯	◯
Successful treatment outcome in hospital setting	◯	◯	◯	◯	◯
Reduction in likelihood of re-offending	◯	◯	◯	◯	◯

Please add your comments, provisos and/or qualifying remarks

4. Are there specific interventions that in your experience, are particularly effective in a hospital setting with offenders who have a diagnosis of DPD? (e.g. psychoeducation, Cognitive Behavioural Therapy, etc.)

* 5. Psychopathy (indicated by a high PCL-R score of 27+):

	Highly likely	Somewhat likely	Somewhat unlikely	Highly unlikely	Unsure
Suitability for hospital admission	◯	◯	◯	◯	◯
Successful treatment outcome in hospital setting	◯	◯	◯	◯	◯
Reduction in likelihood of re-offending	◯	◯	◯	◯	◯

Please add your comments, provisos and/or qualifying remarks

6. Are there specific interventions that in your experience are particularly effective in a hospital setting with offenders who have a diagnosis of Psychopathy? (e.g. psychoeducation, Cognitive Behavioural Therapy, etc.)

Diagnoses Continued

* 1. Multiple co-occuring personality disorders e.g. ASPD and BPD:

	Highly likely	Somewhat likely	Somewhat unlikely	Highly unlikely	Unsure
Suitability for hospital admission	◯	◯	◯	◯	◯
Successful treatment outcome in hospital setting	◯	◯	◯	◯	◯
Reduction in likelihood of re-offending	◯	◯	◯	◯	◯

Please add your comments, provisos and/or qualifying remarks

* 2. Diagnosis of Personality Disorder co-morbid with severe and enduring mental illness (e.g. schizophrenia):

	Highly likely	Somewhat likely	Somewhat unlikely	Highly unlikely	Unsure
Suitability for hospital admission	◯	◯	◯	◯	◯
Successful treatment outcome in hospital setting	◯	◯	◯	◯	◯
Reduction in likelihood of re-offending	◯	◯	◯	◯	◯

Please add your comments, provisos and/or qualifying remarks

* 3. Diagnosis of Personality Disorder co-morbid with mental illness not classified as severe and enduring (e.g. mood disorder without psychotic symptoms):

	Highly likely	Somewhat likely	Somewhat unlikely	Highly unlikely	Unsure
Suitability for hospital admission	◯	◯	◯	◯	◯
Successful treatment outcome in hospital setting	◯	◯	◯	◯	◯
Reduction in likelihood of re-offending	◯	◯	◯	◯	◯

Please add your comments, provisos and/or qualifying remarks

* 4. Diagnosis of Personality Disorder co-morbid with substance misuse/dependence:

	Highly likely	Somewhat likely	Somewhat unlikely	Highly unlikely	Unsure
Suitability for hospital admission	◯	◯	◯	◯	◯
Successful treatment outcome in hospital setting	◯	◯	◯	◯	◯
Reduction in likelihood of re-offending	◯	◯	◯	◯	◯

Please add your comments, provisos and/or qualifying remarks

Diagnoses Continued

1. Are there any personality disorder diagnoses that you consider more amenable to treatment or intervention? If so, please list below:

2. Are there any personality disorder diagnoses that you consider less amenable to treatment? If so, please list below:

Assessing Offenders Transfers from Prison to Forensic Mental Health Services

Characteristics

The following characteristics have been identified by a systematic review of the literature as key features in offenders diagnosed with personality disorder. The below questions ask how specific characteristics impact on a persons suitability for admission to a hospital setting, how they impact on the likelihood of successful treatment outcome (i.e. reduction in symptoms, distress and/or psychopathology) and the likelihood of future re-offending.

* 1. Impulsivity:

	Highly likely	Somewhat likely	Somewhat unlikely	Highly unlikely	Unsure
Suitability for hospital admission	◯	◯	◯	◯	◯
Successful outcome for treatment in hospital	◯	◯	◯	◯	◯
Likely to be a risk factor in future re-offending	◯	◯	◯	◯	◯

Please add your comments, provisos and/or qualifying remarks

* 2. Poor social functioning (i.e. impaired social skills):

	Highly likely	Somewhat likely	Somewhat unlikely	Highly unlikely	Unsure
Suitability for hospital admission	◯	◯	◯	◯	◯
Successful outcome of treatment in hospital	◯	◯	◯	◯	◯
Likely to be a risk factor in future re-offending	◯	◯	◯	◯	◯

Please add your comments, provisos and/or qualifying remarks

* 3. Lack of victim empathy:

	Highly likely	Somewhat likely	Somewhat unlikely	Highly unlikely	Unsure
Suitability for hospital admission	◯	◯	◯	◯	◯
Successful outcome of treatment in hospital	◯	◯	◯	◯	◯
Likely to be a risk factor in future re-offending	◯	◯	◯	◯	◯

Please add your comments, provisos and/or qualifying remarks

Characteristics Continued

* 1. High levels of hostility:

	Highly likely	Somewhat likely	Somewhat unlikely	Highly unlikely	Unsure
Suitability for hospital admission	○	○	○	○	○
Successful outcome of treatment in hospital	○	○	○	○	○
Likely to be a risk factor in future re-offending	○	○	○	○	○

Please add your comments, provisos and/or qualifying remarks

* 2. High levels of interpersonal aggression:

	Highly likely	Somewhat likely	Somewhat unlikely	Highly unlikely	Unsure
Suitability for hospital admission	○	○	○	○	○
Successful outcome of treatment in hospital	○	○	○	○	○
Likely to be a risk factor in future re-offending	○	○	○	○	○

Please add your comments, provisos and/or qualifying remarks

* 3. High levels of institutional violence:

	Highly likely	Somewhat likely	Somewhat unlikely	Highly unlikely	Unsure
Suitability for hospital admission	○	○	○	○	○
Successful outcome of treatment in hospital	○	○	○	○	○
Likely to be a risk factor in future re-offending	○	○	○	○	○

Please add your comments, provisos and/or qualifying remarks

* 4. Individuals who have a negative relationship with their personality disorder diagnosis:

	Highly likely	Somewhat likely	Somewhat unlikely	Highly unlikely	Unsure
Suitability for hospital admission	◯	◯	◯	◯	◯
Successful outcome of treatment in hospital	◯	◯	◯	◯	◯
Likely to be a risk factor in future re-offending	◯	◯	◯	◯	◯

Please add your comments, provisos and/or qualifying remarks

Characteristics Continued

The following characteristics have been identified by a systematic review of the literature as key features in offenders diagnosed with personality disorder. The below questions ask how specific characteristics impact on a persons suitability for admission to a hospital setting, how they impact on the likelihood of successful treatment outcome (i.e. reduction in symptoms, distress and/or psychopathology) and the likelihood of future re-offending.

* 1. IQ score below 90:

	Highly likely	Somewhat likely	Somewhat unlikely	Highly unlikely	Unsure
Suitability for hospital admission	◯	◯	◯	◯	◯
Successful outcome of treatment in hospital	◯	◯	◯	◯	◯
Likely to be a risk factor in future re-offending	◯	◯	◯	◯	◯

Please add your comments, provisos, and/or qualifying remarks

* 2. Aged under 35 years old:

	Highly likely	Somewhat likely	Somewhat unlikely	Highly unlikely	Unsure
Suitability for hospital admission	◯	◯	◯	◯	◯
Successful outcome of treatment in hospital	◯	◯	◯	◯	◯
Likely to be a risk factor in future re-offending	◯	◯	◯	◯	◯

Please add your comments, provisos, and/or qualifying remarks

* 3. Previous psychiatric history (e.g. a past diagnosis of severe mental illness or inpatient psychiatric admission):

	Highly likely	Somewhat likely	Somewhat unlikely	Highly unlikely	Unsure
Suitability for admission to a hospital setting	◯	◯	◯	◯	◯
likelihood of successful treatment outcome	◯	◯	◯	◯	◯
Likely to be a risk factor in future re-offending	◯	◯	◯	◯	◯

If so, how does it impact?

* 4. Suicidal ideation:

	Highly likely	Somewhat likely	Somewhat unlikely	Highly unlikely	Unsure
Suitability for admission to a hospital setting	◯	◯	◯	◯	◯
Likelihood of successful treatment outcome	◯	◯	◯	◯	◯
Likely to be a risk factor in future re-offending	◯	◯	◯	◯	◯

Please add your comments, provisos and/or qualifying remarks

Characteristics Continued

The following characteristics have been identified by a systematic review of the literature as key features in offenders diagnosed with personality disorder. The below questions ask how specific characteristics impact on a persons suitability for admission to a hospital setting, how they impact on the likelihood of successful treatment outcome (i.e. reduction in symptoms, distress and/or psychopathology) and the likelihood of future re-offending.

Please note, when answering the last question on the matrix scales 'Likely to be a risk factor in future re-offending' ticking 'highly/somewhat likely' would indicate an increase in the risk of re-offending.

* 1. Conscientious individuals:

	Highly likely	Somewhat likely	Somewhat unlikely	Highly unlikely	Unsure
Suitability for hospital admission	○	○	○	○	○
Successful outcome of treatment in hospital	○	○	○	○	○
Likely to be a risk factor in future re-offending	○	○	○	○	○

Please add your comments, provisos and/or qualifying remarks

* 2. Extroverted individuals:

	Highly likely	Somewhat likely	Somewhat unlikely	Highly unlikely	Unsure
Suitability for hospital admission	○	○	○	○	○
Successful outcome of treatment in hospital	○	○	○	○	○
Likely to be a risk factor in future re-offending	○	○	○	○	○

Please add your comments, provisos and/or qualifying remarks

* 3. Higher levels of perceived motivation for treatment:

	Highly likely	Somewhat likely	Somewhat unlikely	Highly unlikely	Unsure
Suitability for hospital admission	○	○	○	○	○
Successful outcome of treatment in hospital	○	○	○	○	○
Likely to be a risk factor in future re-offending	○	○	○	○	○

Please add your comments, provisos and/or qualifying remarks

Assessing Offenders Transfers from Prison to Forensic Mental Health Services

Offence history

Offence history was identified as a contributing factor when considering the management and treatment of offenders with personality disorder. The below questions ask more specifically of how a person's offence history impact on a persons suitability for admission to a hospital setting, the likelihood of successful treatment outcome (i.e. reduction in symptoms, distress and/or psychopathology) and the likelihood of future re-offending.

* 1. Previous offending and/or convictions:

	Highly likely	Somewhat likely	Somewhat unlikely	Highly unlikely	Unsure
Suitability for hospital admission	○	○	○	○	○
Successful outcome of treatment in hospital	○	○	○	○	○
Likely to be a risk factor in future re-offending	○	○	○	○	○

Please add your comments, provisos, and/or qualifying remarks

* 2. Early onset offending (aged under 18 years old):

	Highly likely	Somewhat likely	Somewhat unlikely	Highly unlikely	Unsure
Suitability for hospital admission	○	○	○	○	○
Successful outcome of treatment in hospital	○	○	○	○	○
Likely to be a risk factor in future re-offending	○	○	○	○	○

Please add your comments, provisos, and/or qualifying remarks

Offence history continued

* 1. Having had 10 or more convictions before reaching the age of 18:

	Highly likely	Somewhat likely	Somewhat unlikely	Highly unlikely	Unsure
Suitability for hospital admission	○	○	○	○	○
Successful outcome of treatment in hospital	○	○	○	○	○
Likely to be a risk factor in future re-offending	○	○	○	○	○

Please add your comments, provisos and/or qualifying remarks

* 2. Previous attendance at any appropriate community supervision (e.g. through health services or probation):

	Highly likely	Somewhat likely	Somewhat unlikely	Highly unlikely	Unsure
Suitability for hospital admission	○	○	○	○	○
Successful outcome of treatment in hospital	○	○	○	○	○
Likely to be a risk factor in future re-offending	○	○	○	○	○

Please add your comments, provisos and/or qualifying remarks

* 3. Sexual index offence:

	Highly likely	Somewhat likely	Somewhat unlikely	Highly unlikely	Unsure
Suitability for admission to hospital	○	○	○	○	○
Successful outcome of treatment in hospital	○	○	○	○	○
Likely to be a risk factor in future re-offending	○	○	○	○	○

Please add your comments, provisos, and/or qualifying remarks

* 4. Violent index offence:

	Highly likely	Somewhat likely	Somewhat unlikely	Highly unlikely	Not sure
Suitability for hospital admission	◯	◯	◯	◯	◯
Successful outcome of treatment in hospital	◯	◯	◯	◯	◯
Likely to be a risk factor in future re-offending	◯	◯	◯	◯	◯

Please add your comments, provisos and/or qualifying remarks

Other

The below questions ask you to consider an individuals engagement with previous treatment, thinking about how this impacts on the following: suitability for admission to a hospital setting; the likelihood of successful treatment outcome (i.e. reduction in symptoms, distress and/or psychopathology) and the likelihood of future re-offending.

Please note, when answering the last question on the matrix scales 'Likely to be a risk factor in future re-offending' ticking 'highly/somewhat likely' would indicate that previous treatment is likely to increase in the risk of re-offending.

* 1. Completion of any previous psychological treatment programmes in prison (e.g. treatment in a DSPD service):

	Highly likely	Somewhat likely	Somewhat unlikely	Highly unlikely	Unsure
Suitability for hospital admission	○	○	○	○	○
Successful outcome of treatment in hospital	○	○	○	○	○
Likely to be a risk factor in future re-offending	○	○	○	○	○

Please add your comments, provisos, and/or qualifying remarks

* 2. Completion of any previous psychological treatment programmes elsewhere (e.g. in the community):

	Highly likely	Somewhat likely	Somewhat unlikely	Highly unlikely	Unsure
Suitability for admission to hospital	○	○	○	○	○
Successful outcome of treatment in hospital	○	○	○	○	○
Likely to be a risk factor in future re-offending	○	○	○	○	○

Please add your comments, provisos, and/or qualifying remarks

Assessing Offenders Transfers from Prison to Forensic Mental Health Services

Other

* 1. Presence of severe and enduring mental illness, managed by appropriate medication:

	Highly likely	Somewhat likely	Somewhat unlikely	Highly unlikely	Unsure
Suitability for hospital admission	○	○	○	○	○
Successful outcome of treatment in hospital	○	○	○	○	○
Likely to be a risk factor in future re-offending	○	○	○	○	○

Please add your comments, provisos, and/or qualifying remarks

* 2. Once receiving treatment in a hospital setting, how does the therapeutic relationship impact on:

	Not at all	Mildly	Moderately	Severely	Unsure
Successful outcome of hospital treatment	○	○	○	○	○
Reduction in likelihood of re-offending	○	○	○	○	○

If so, can you expand on this further:

* 3. What staff qualities are important when working with offenders with personality disorder who are admitted to a hospital setting?

1)

2)

3)

4)

5)

* 4. Treatment in a forensic mental health unit is most likely to be appropriate for offenders with what level risk for violent and/or sexual re-offending? (check all that apply)

- [ ] Low risk
- [ ] Medium risk
- [ ] High risk
- [ ] Very high risk
- [ ] Please add your comments, provisos and/or qualifying remarks

* 5. For those offenders admitted to hospital, what is the optimum length of stay over which they will benefit most from intervention?

- [ ] Less than 1 year
- [ ] 1-2 years
- [ ] 2-3 years
- [ ] 3-4 years
- [ ] 4+ years
- [ ] Please add your comments, provisos and/or qualifying remarks

6. What assessment tools do you routinely use to assess progress and treatment outcomes?

- [ ] HCR 20
- [ ] SVR-20
- [ ] IPDE Dimensions
- [ ] PDQ-4
- [ ] IORS
- [ ] PAI
- [ ] HONOS-S
- [ ] Case formulation

Please state any other tools your routinely use:

1. Please list any other important factors which, in your experience, suggest a transfer to hospital is indicated. This should not be because a suitable prison-based service is full, but because treatment in hospital stands a better chance of reducing distress, an/or psychopathology, and/or risk. We would encourage you to think broadly, and include any factors in any domain(s) that you feel to be important, e.g. demographic, clinical, social, physical, institutional, risk-related (to self or others) or combinations of these. Alternatively, you might feel that no additional factors are important in this regard.

Factor 1

Factor 2

Factor 3

Factor 4

Factor 5

Factor 6

Factor 7

Factor 8

Thank You

Thank you for taking the time to complete this survey, your response has now been recorded. We truly value the information you have provided.

As this survey is the first part of a two-stage Delphic process the responses will now be synthesised to create the second part of the survey where contact will be made with you again.

If you have any comments on the survey, please contact Zoe Foyston,
z.n.z.goodwin@smd16.qmul.ac.uk
Once again, thank you.

1. Please check off below if you do NOT want to be contacted to complete the second survey

◯ Please do not contact me
